# Supplementary material for: Bioactive Nitrosylated and Nitrated N-(2-hydroxyphenyl)acetamides and Derived Oligomers: An Alternative Pathway to 2-Amidophenol-Derived Phytotoxic Metabolites
Source: Molecules. 2022 Jul 26;27(15):4786. doi: 10.3390/molecules27154786 (PMC9330447; doi:10.3390/molecules27154786)
Supplement: Supplementary file 1 [file molecules-27-04786-s001.zip › molecules-1805926-supplementary.pdf]

Supplementary File to

Bioactive nitrosylated and nitrated N-(2-Hydroxyphenyl)acetamides and Derived Oligomers: An Alternative Pathway to 2-Amidophenol Derived Phytotoxic Metabolites

Sergey Girel 1, Vadim Schütz 2, Laurent Bigler 1, *, Peter Dörmann 2 and Margot Schulz 2,*

Supplementary Figures

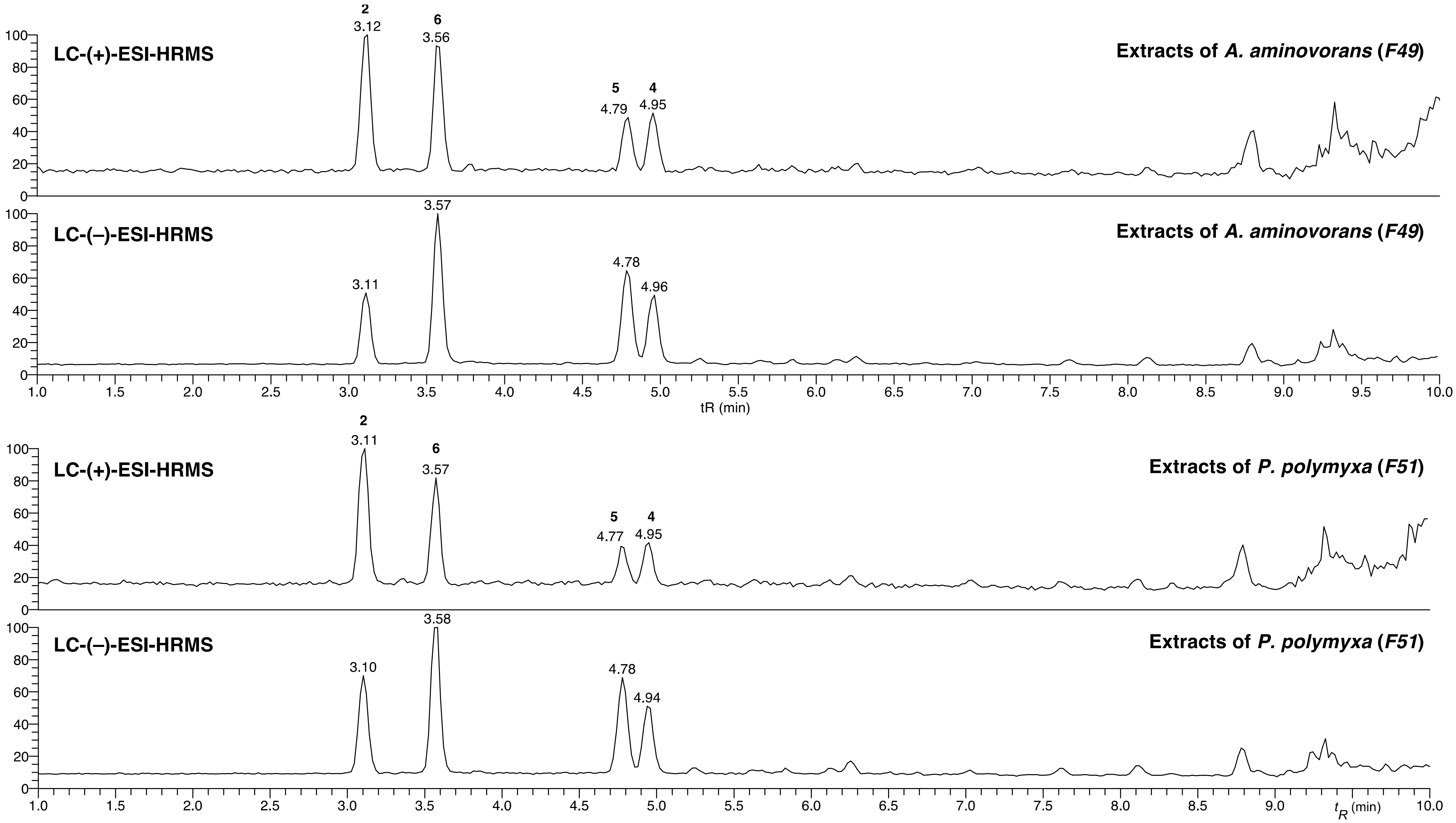

Figure S1. LC/HRMS chromatograms of bacterial exudate extracts from *A. aminovorans* and *P. Polymyxa* in the positive and negative ionization mode

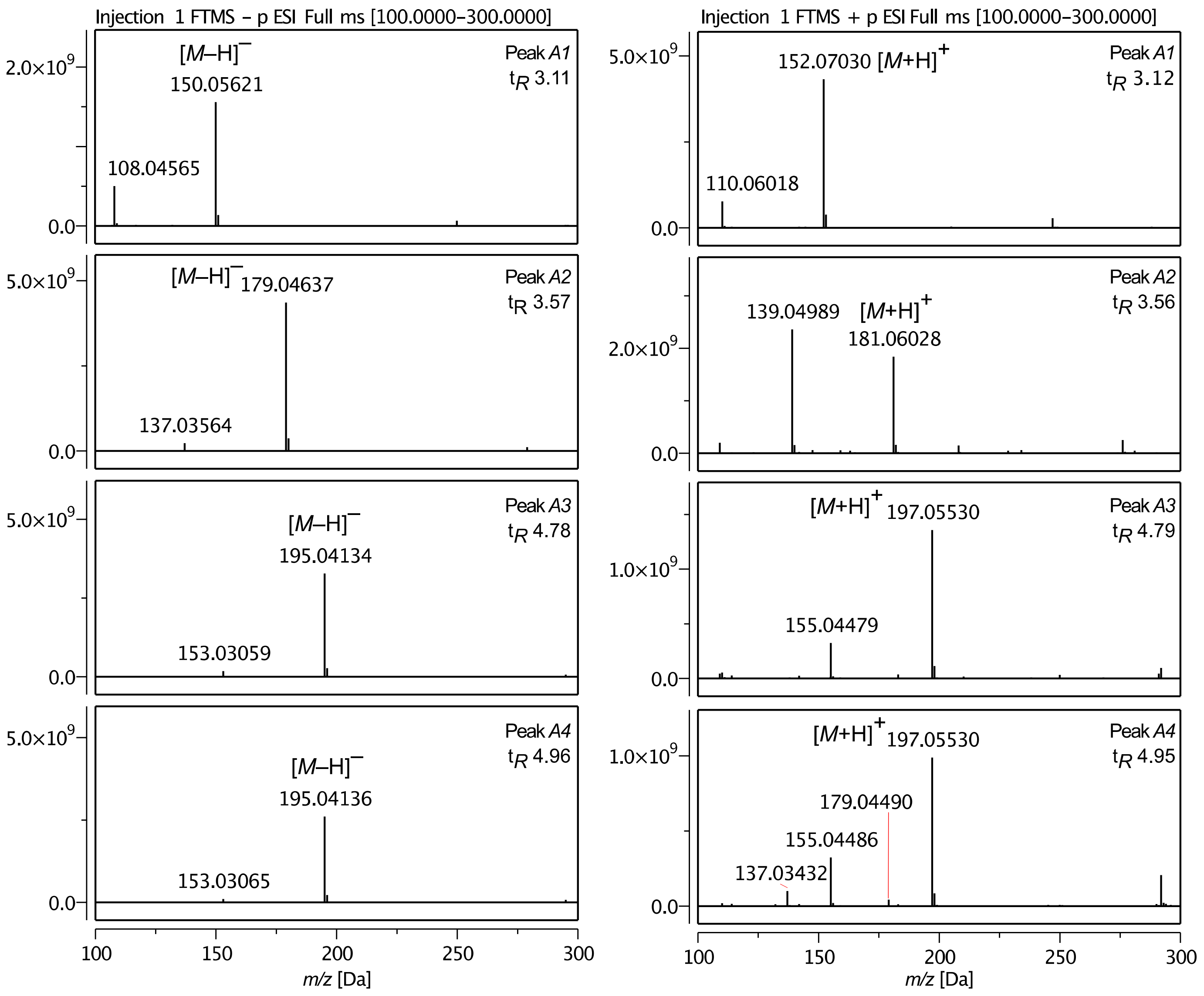

Figure S2. (–)- and (+)-ESI HR-MS of the chromatographic peaks *A1-A4* obtained from the bacterial culture extract *F49*

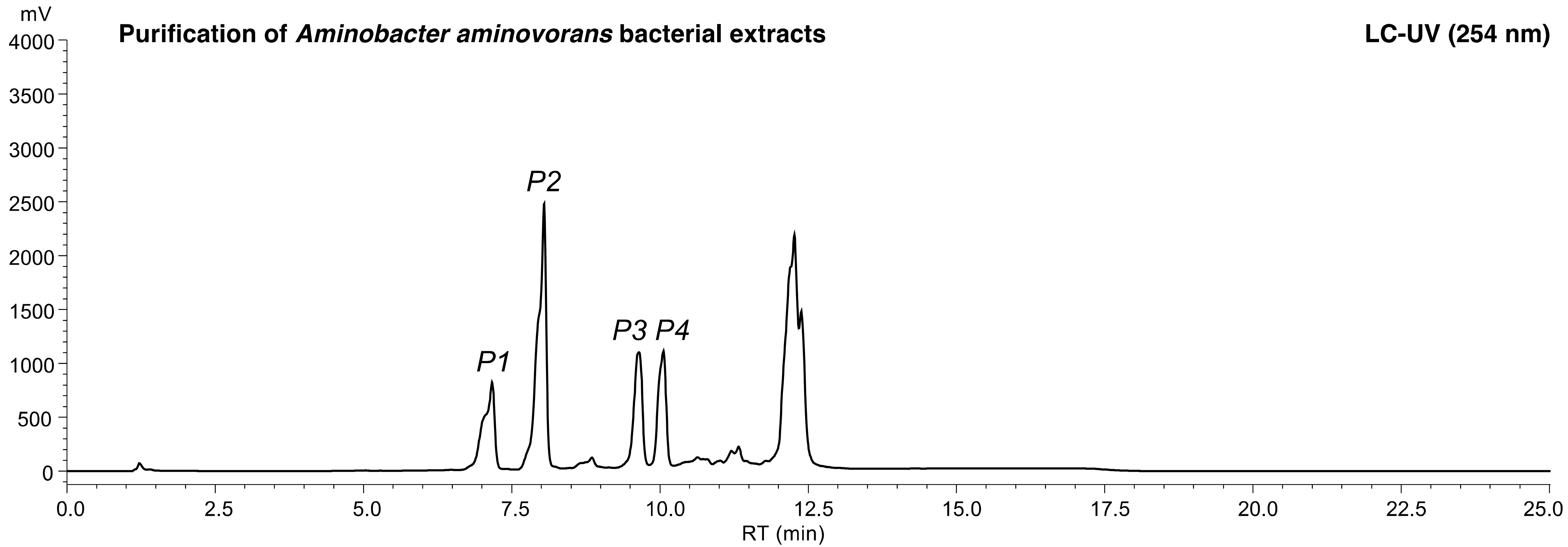

Figure S3. Preparative LC-UV of bacterial extracts from *A. aminovorans* showing the four fractions that were collected for structure elucidation. Chromatographic conditions: *Waters Cortecs* column (C18, 4.6x100mm, 2.7 um)

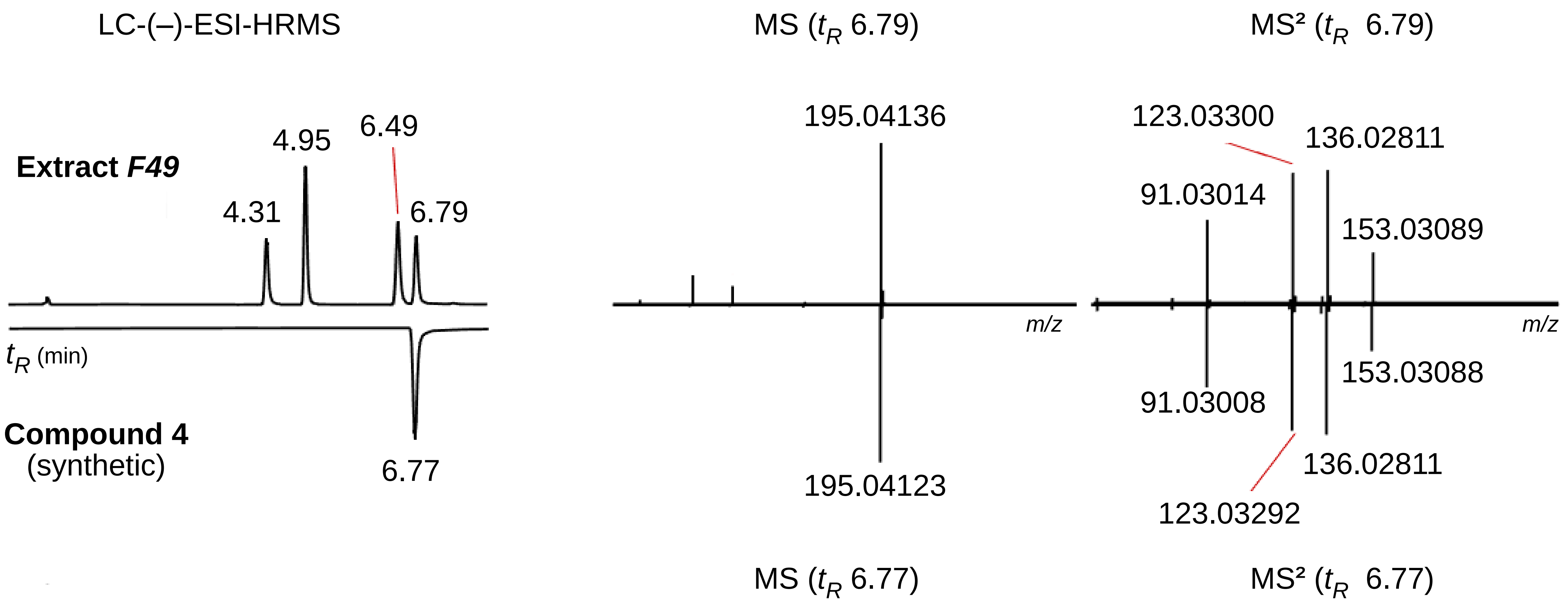

Figure S4. LC-(–)-ESI-HR-MS(/MS) confirmation of the *N*-(2-hydroxy-3-nitrophenyl)acetamide structure **4** isolated from bacterial exudate extracts of *A. aminovorans* (corresponding to fraction *P4*). Chromatographic conditions: *Waters ACQUITY HSS T3* column (C18, 2.1x100mm, 1.8 um, 0.4 mL/min, H_2O/CH_3CN + HCOOH 0.1%, gradient elution).

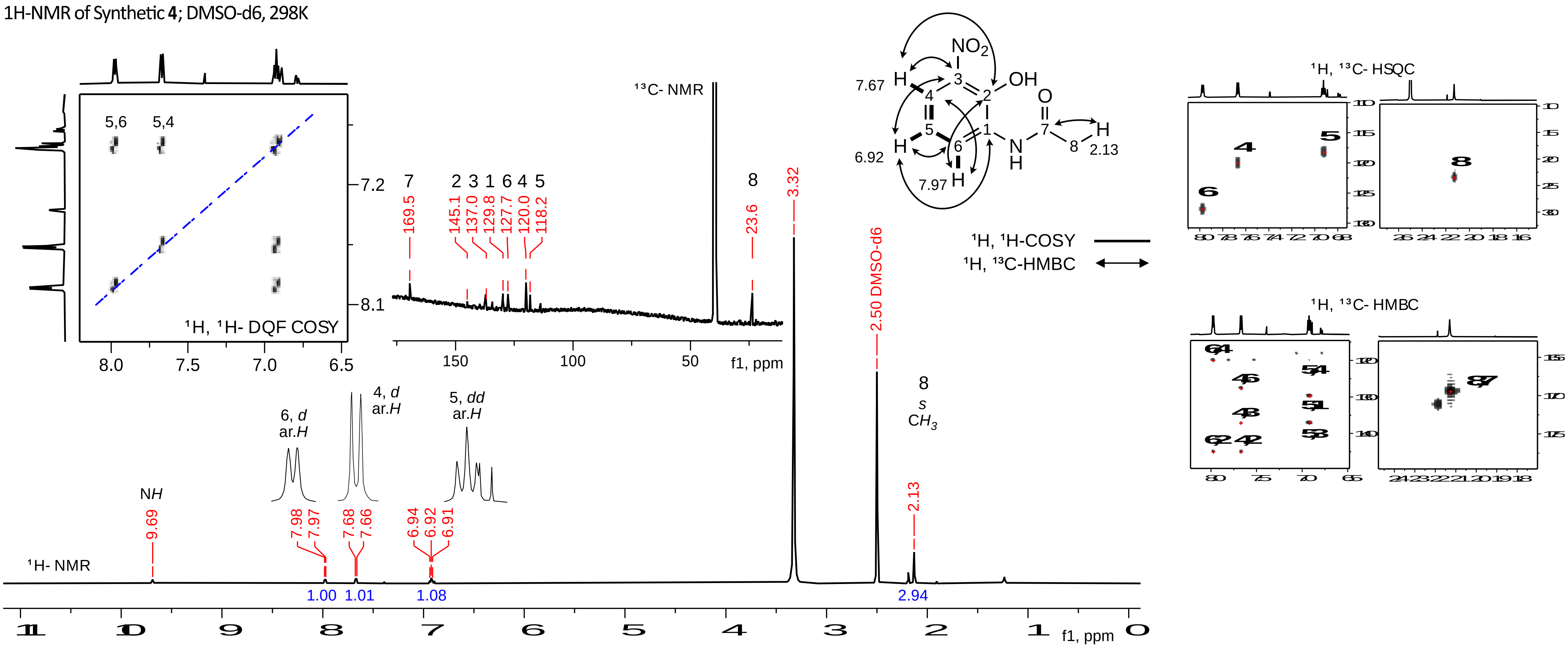

Figure S5. 1D- & 2D-NMR data of synthetic compound **4**. The *dd* at 6.92 ppm coupled with the doublets at 7.97 and 7.67 ppm, indicating the NO_2 group being either on position C^3 or C^5 (numbering according to ^{13}C-NMR). The positions of OH and NO_2 groups were determined by comparing the chemical shifts of the corresponding quaternary carbons with those of the commercial substance **5**.

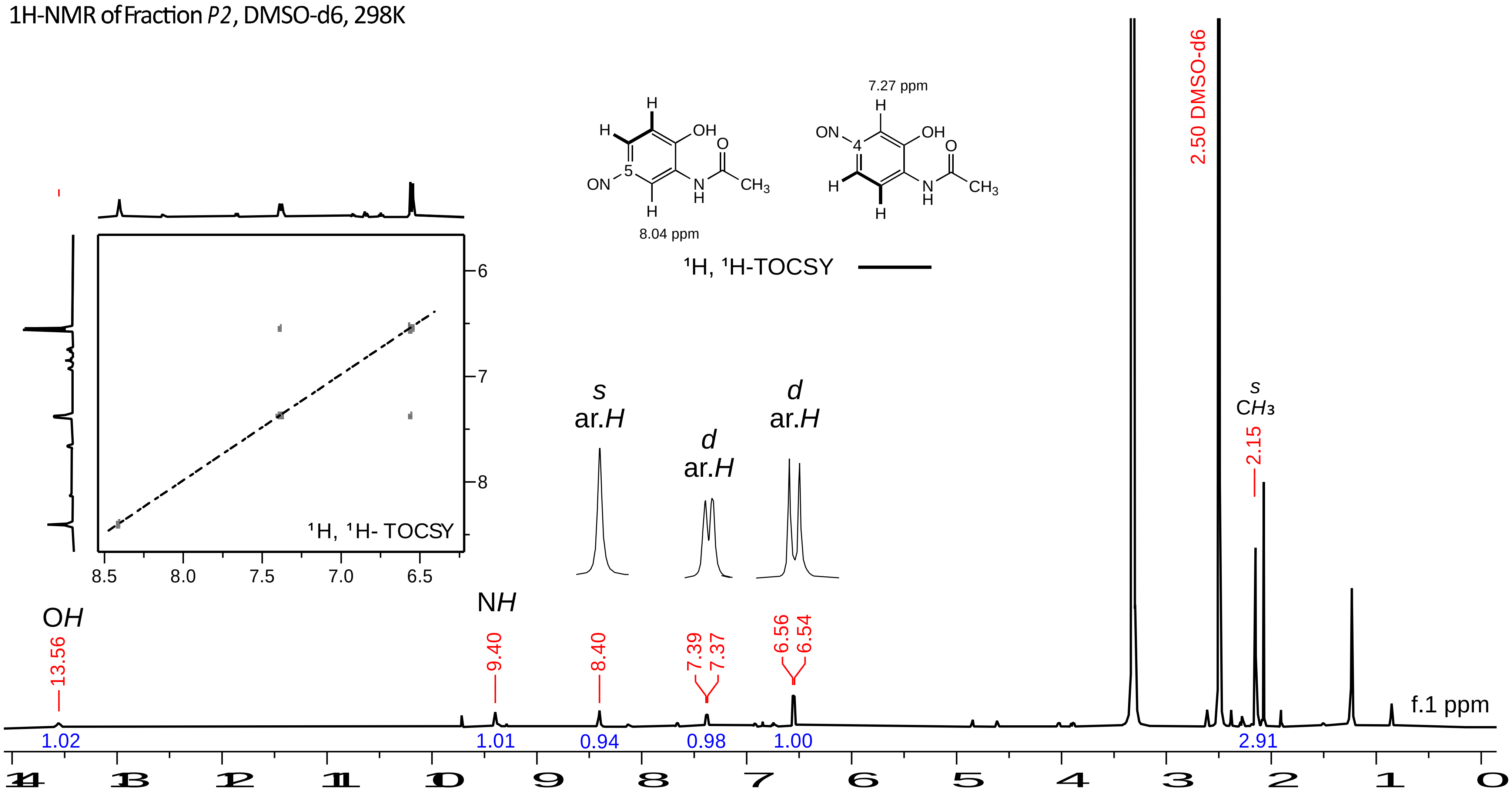

Figure S6. ^{1}H- & TOCSY NMR data of fraction *P2* (**6**) obtained after purification of the bacterial exudate extracts of *A. aminovorans*. The doublet at 7.38 ppm coupling with the doublet at 6.55 ppm indicates substitution of the aromatic motif at position C^4 or C^5. Further, singlets at 13.56 and 2.15 ppm represent phenolic OH proton and methyl protons of *N*-acetyl motif.

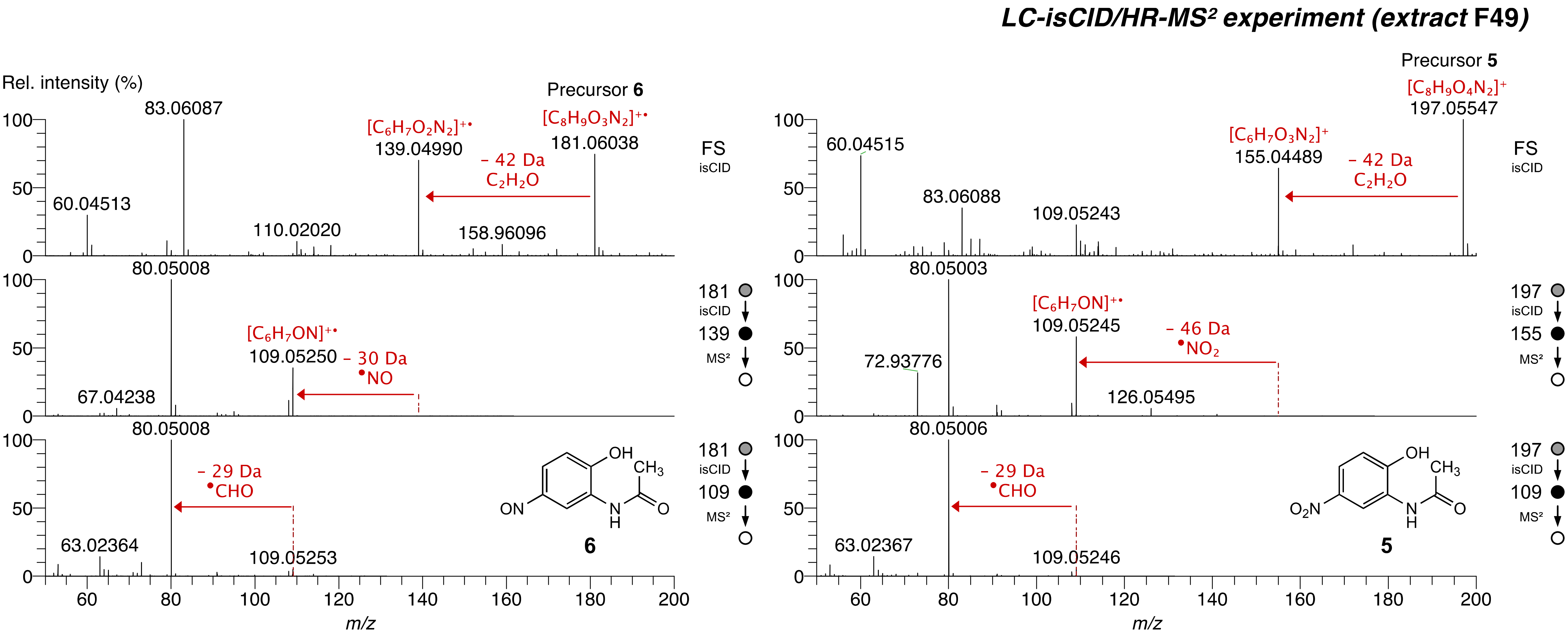

	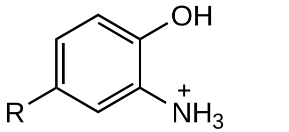	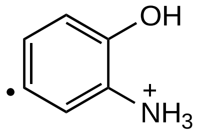	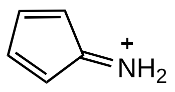
R = NO	*m/z* 139.04990, -0.3 mDa	*m/z* 109.05250, +0.28 mDa	*m/z* 80.05008, +0.6 mDa
R = NO_2	*m/z* 155.04489, -0.23 mDa	*m/z* 109.05245, +0.23 mDa	*m/z* 80.05006, +0.6 mDa

Figure S7. LC-(+)-ESI-in-source CID/HR-MS^2 spectra of **5** (right) and **6** (left) obtained from the *F49* bacterial extract. The loss of a $NO^{\bullet}$ radical following the cleavage of ethenone (*m/z* 181 > 139 > 109, left) can be compared to the loss of the $NO_2^{\bullet}$ radical from the same position (right).

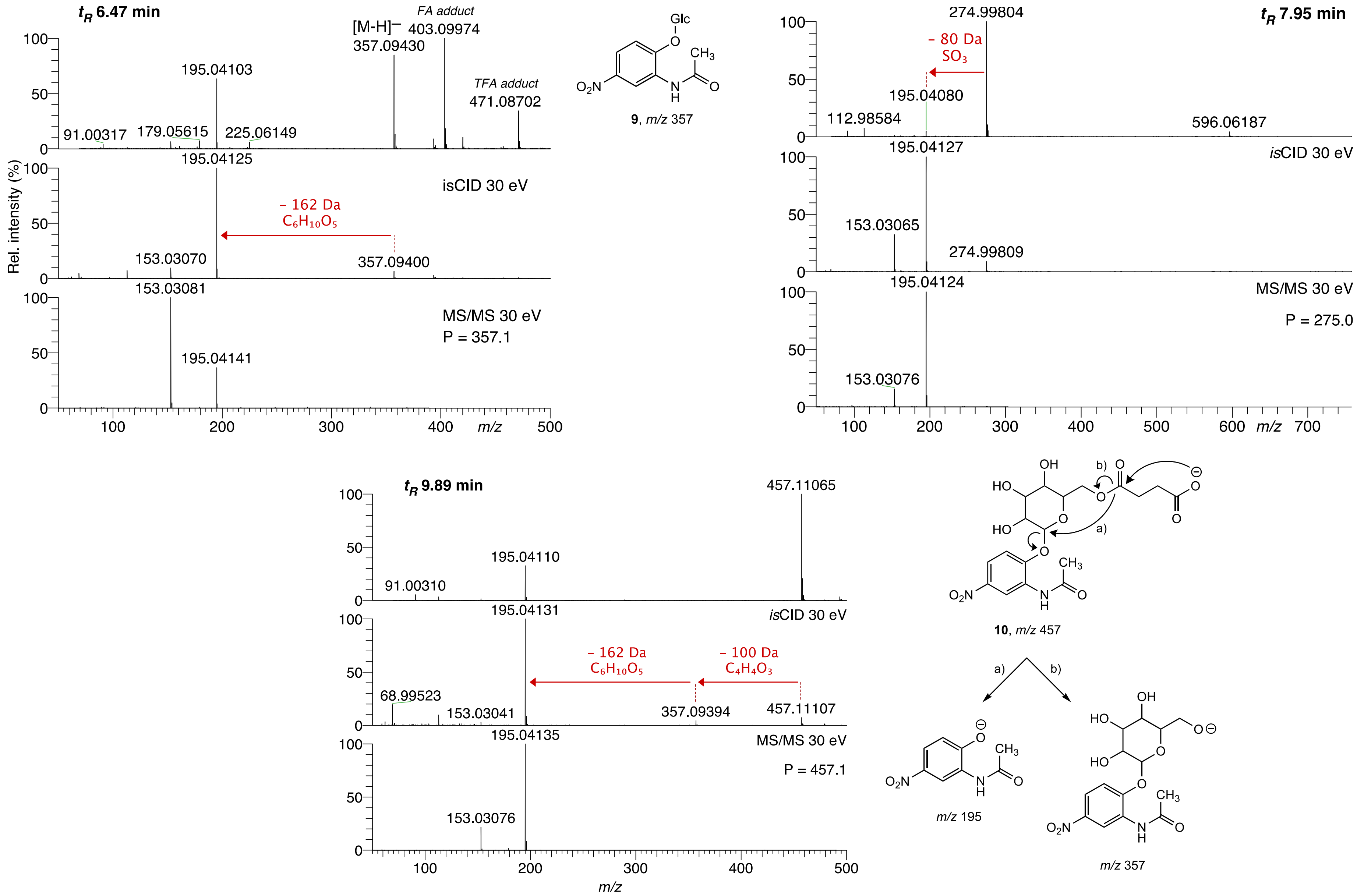

Figure S8. LC-(+)-ESI-in-source CID/HR-MS^2 spectra and structure elucidation of the metabolites detected and annotated in the bacterial extract of *A. aminovorans F49*, in bacterial and fungal extracts after incubation with *N*-(2-hydroxy-5-nitrophenyl)acetamide (**5**)

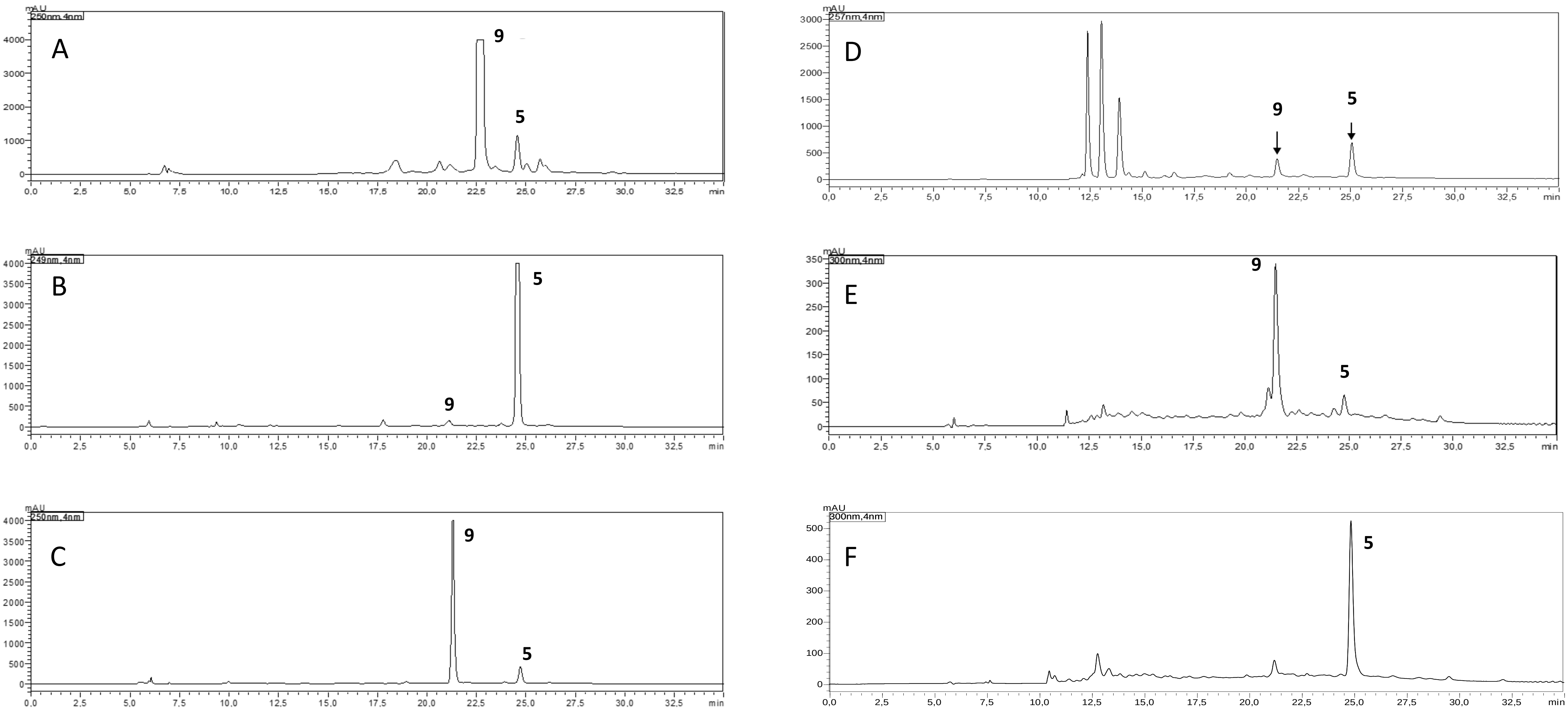

Figure S9: Deglucosylation of **9** by *Arabidopsis* and microorganisms. (A) HPLC Chromatogram of a methanolic root extract prepared from *Arabidopsis thaliana* after 48h incubation with 1 mM *N*-(2-hydroxy-5-nitrophenyl)acetamide **(5)** contains high amounts of **9**. (B) Chromatogram of the incubation of **9** with β-glucosidase 1 min after start, (C) chromatogram after 45 min of incubation. Almost all glucoside is hydrolyzed to **5**. (D) Incubation of *Pantoea ananatis* with **9**. After 30h, deglucosylation of **5** is almost complete. (E) Incubation of *Pseudomonas laurentiana* with **9**, start of the incubation. (F) Incubation after 36 h. Almost all **9** is deglucosylated to **5**.

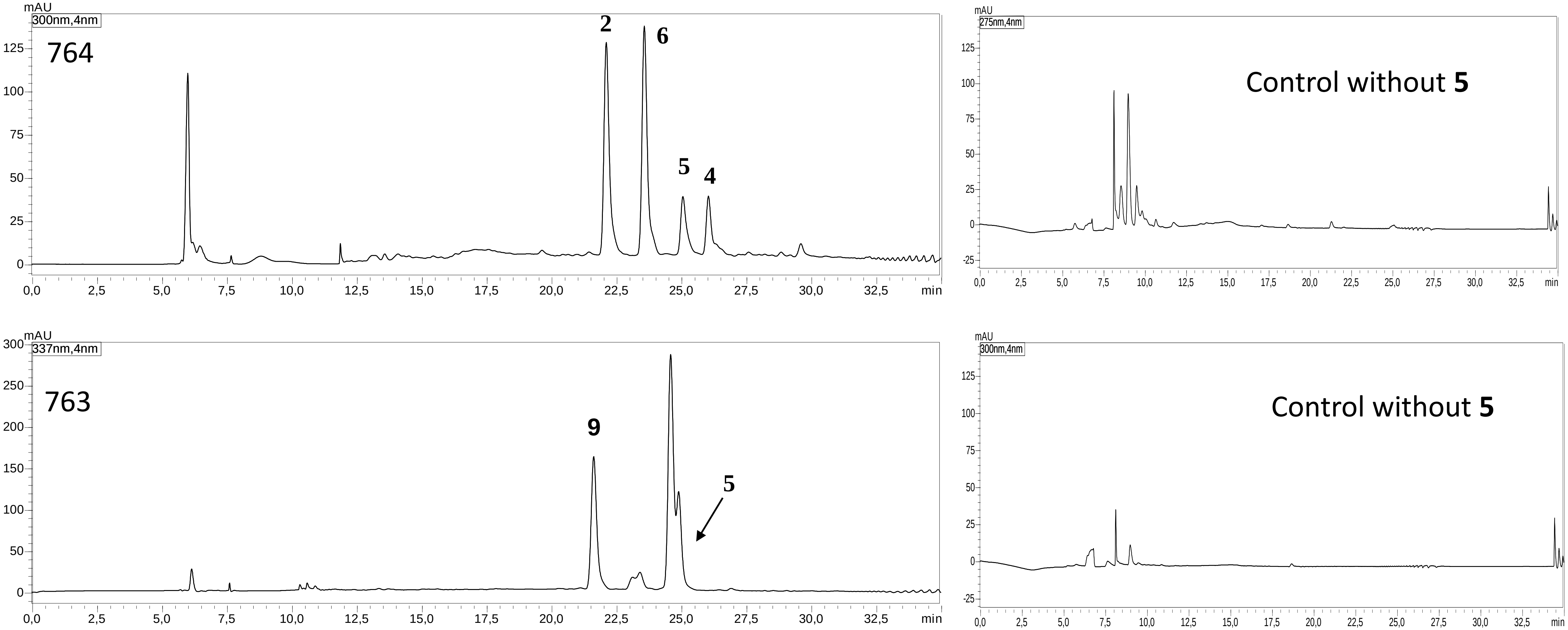

Figure S10: *Arthrobacter* species MPI 764 metabolized 2-acetamido-phenol **(2),** yielding **4**, **5** and **6**. *Arthrobacter* species MPI 763 glucosylated **5** to **9**. The compounds are present in the culture mediums when supplemented with **2** (MPI 764); with **5** (MPI 763). On the right side the controls of the corresponding incubations without **5** are shown. The *Arthrobacter* species do not contain **5** or any related compound.

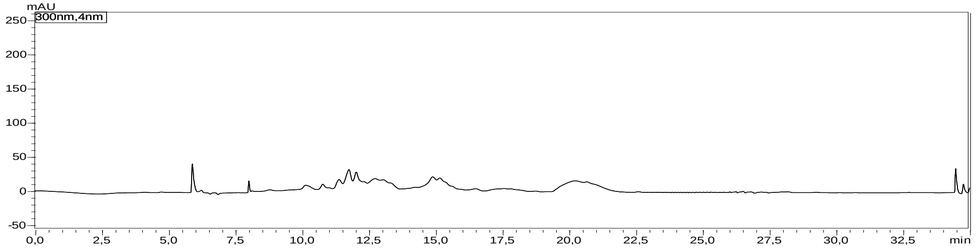

Kohlrabi control exctract from roots incubation without (5)

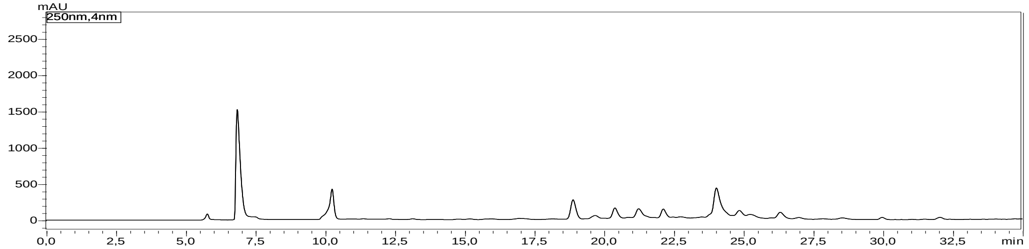

Arabidopsis control exctract from roots incubation without (5)

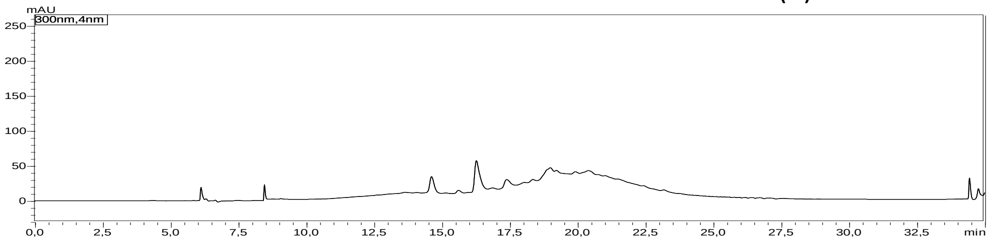

Aminobacter aminovorans control incubation, medium without (5)

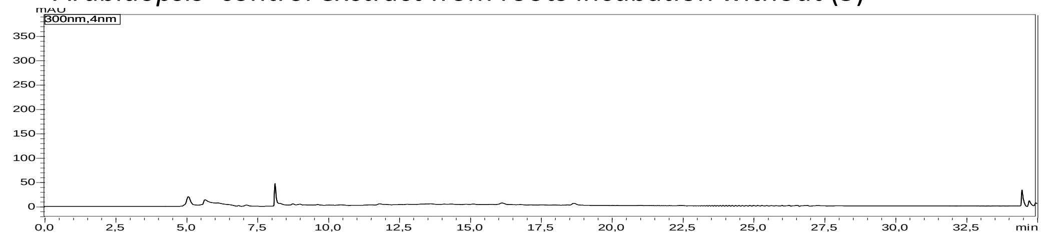

Pseudomonas laurentiana control incubation, medium without (5)

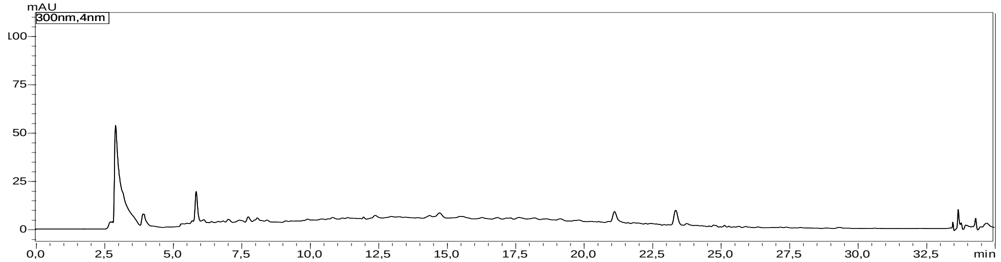

Pantoea ananatis control incubation, medium without (5)

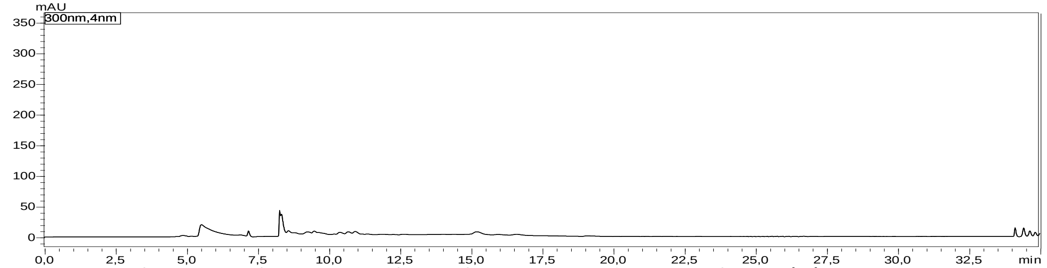

Papiliotrema baii control incubation, medium without (5)

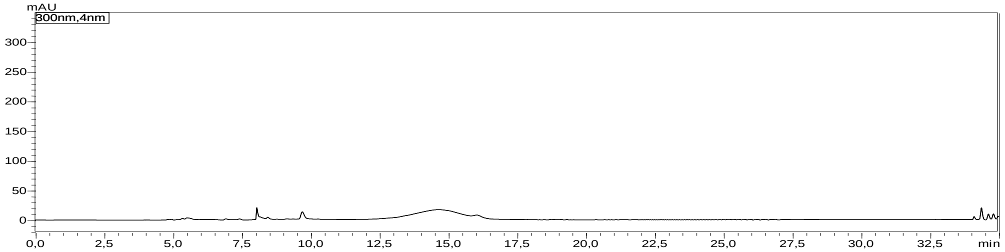

Actinomucor elegans consortium control incubation, medium without (5)

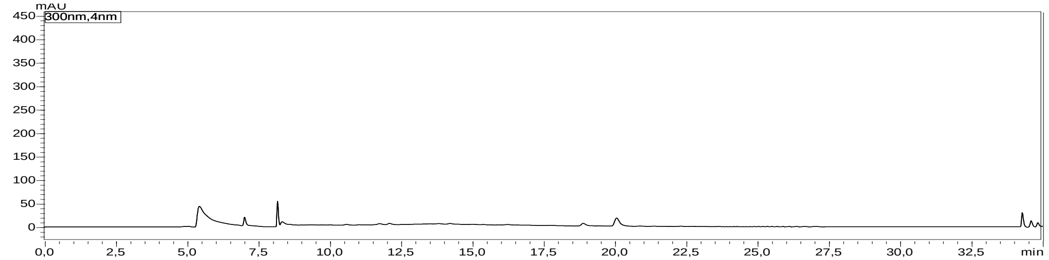

Trichoderma viride consortium control incubation, medium without (5)

Figure S11: Chromatograms of extracts from plants and microorganisms incubated without **5** as controls. None of the control extracts contained **5** or any related compound (compare Fig. S9).

Supplementary Tables

t_R	**Name**	**Structure**	**Source**
4.31	*N*-(2-Hydroxy-6-nitrophenyl)acetamide **(7)**	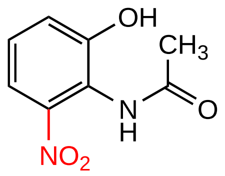	Synthetic (see exp. part)
6.49	*N*-(2-Hydroxy-5-nitrophenyl)acetamide **(5)**	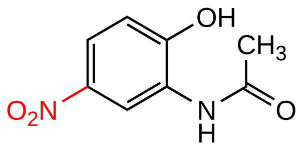	Commercial
6.77	*N*-(2-Hydroxy-3-nitrophenyl)acetamide **(4)**	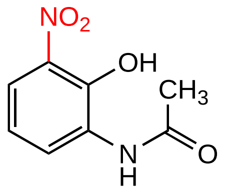	Synthetic (see exp. part)
7.30	*N*-(2-Hydroxy-4-nitrophenyl)acetamide **(8)**	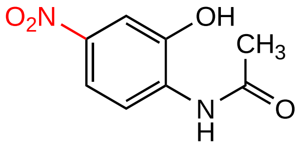	Commercial

Table S1. Chromatographic separation of the four nitrophenyl-derivatives of 2-acetamido-phenol. Chromatographic conditions: *Waters ACQUITY HSS T3* column (C18, 2.1x100mm, 1.8 um)

t_R	Peak #	Metabolite	Structure	Neutral formula	Ion ESI-	*m/z* calc	*m/z* meas	Err., mDa	Ion ESI+	*m/z* calc	*m/z* meas	Err., mDa
3.36	D1	Dimer	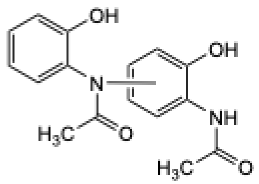	$C_{16}H_{16}N_2O_4$	$[M+H]^-$	299.10373	299.10354	-0.19	$[M+H]^+$	301.1177	301.11869	0.99
5.64	D2			$C_{16}H_{16}N_2O_4$	$[M+H]^-$	299.10373	299.10355	-0.18	$[M+H]^+$	301.1177	301.11779	0.09
6.71	D3			$C_{16}H_{16}N_2O_4$	$[M+H]^-$	299.10373	299.10357	-0.16	$[M+H]^+$	301.1177	301.11778	0.08
6.97	D4			$C_{16}H_{16}N_2O_4$	$[M+H]^-$	299.10373	299.10360	-0.13	$[M+H]^+$	301.1177	301.11760	-0.10
5.26	D5	Dimer [+ NO]	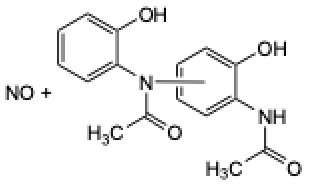	$C_{16}H_{15}N_3O_5$	$[M+H]^-$	328.09389	328.09375	-0.14	$[M+H]^+$	330.10845	330.10798	-0.47
5.71	D6			$C_{16}H_{15}N_3O_5$	$[M+H]^-$	328.09389	328.09383	-0.06	$[M+H]^+$	330.10845	330.10788	-0.57
6.26	D7			$C_{16}H_{15}N_3O_5$	$[M+H]^-$	328.09389	328.09344	-0.45	$[M+H]^+$	330.10845	330.10782	-0.63
8.12	D8			$C_{16}H_{15}N_3O_5$	$[M+H]^-$	328.09389	328.09368	-0.21	$[M+H]^+$	330.10845	330.10815	-0.30
8.14	D9	Dimer [+ NO_2]	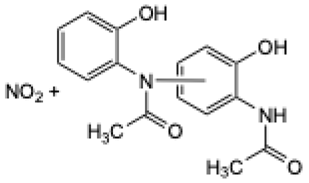	$C_{16}H_{15}N_3O_6$	$[M+H]^-$	344.08826	344.08855	0.29	$[M+H]^+$	346.10336	346.10305	-0.31
8.80	D10			$C_{16}H_{15}N_3O_6$	$[M+H]^-$	344.08826	344.08839	0.13	$[M+H]^+$	346.10336	346.10281	-0.55
9.09	D11			$C_{16}H_{15}N_3O_6$	$[M+H]^-$	344.08826	344.08877	0.51	$[M+H]^+$	346.10336	346.10306	-0.30

Table S2. List of the 2-acetamido-phenol dimers and their nitrosylated and nitrated derivatives detected in the bacterial exudate extracts *F49* of *A. aminovorans*. The corresponding chromatogram is shown on figure 4.

t$_R$	Peak #	Metabolite	Structure	Neutral formula	Ion ESI-	*m/z* calc	*m/z* meas	Err., mDa	Ion ESI+	*m/z* calc	*m/z* meas	Err., mDa
7.01	T1	Trimer	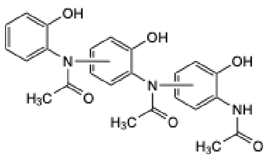	$C_{24}H_{23}N_3O_6$	$[M+H]^-$	448.15141	448.15151	0.10	$[M+H]^+$	450.16596	450.16499	-0.97
7.24	T2			$C_{24}H_{23}N_3O_6$	$[M+H]^-$	448.15141	448.15196	0.55	$[M+H]^+$	450.16596	450.16487	-1.09
7.37	T3			$C_{24}H_{23}N_3O_6$	$[M+H]^-$	448.15141	448.15210	0.69	$[M+H]^+$	450.16596	450.16525	-0.71
7.69	T4			$C_{24}H_{23}N_3O_6$	$[M+H]^-$	448.15141	448.15158	0.17	$[M+H]^+$	450.16596	450.16481	-1.15
7.95	T5			$C_{24}H_{23}N_3O_6$	$[M+H]^-$	448.15141	448.15214	0.73	$[M+H]^+$	450.16596	450.165	-0.96
8.26	T6			$C_{24}H_{23}N_3O_6$	$[M+H]^-$	448.15141	448.15196	0.55	$[M+H]^+$	450.16596	450.16519	-0.77
5.96	T7	Trimer [+ NO]	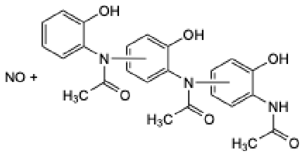	$C_{24}H_{22}N_4O_7$	$[M+H]^-$	477.14157	477.14177	0.20	$[M+H]^+$	479.15613	479.15587	-0.26
6.42	T8			$C_{24}H_{22}N_4O_7$	$[M+H]^-$	477.14157	477.14188	0.31	$[M+H]^+$	479.15613	479.15604	-0.09
6.79	T9			$C_{24}H_{22}N_4O_7$	$[M+H]^-$	477.14157	477.14158	0.01	$[M+H]^+$	479.15613	479.15571	-0.42
7.29	T10			$C_{24}H_{22}N_4O_7$	$[M+H]^-$	477.14157	477.14163	0.06	$[M+H]^+$	479.15613	479.15492	-1.21
7.52	T11			$C_{24}H_{22}N_4O_7$	$[M+H]^-$	477.14157	477.14221	0.64	$[M+H]^+$	479.15613	479.15611	-0.02
7.67	T12	Trimer [+ NO_2]	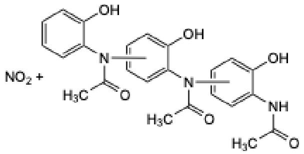	$C_{24}H_{22}N_4O_8$	$[M+H]^-$	493.13649	493.13700	0.51	$[M+H]^+$	495.15104	495.14996	-1.08
7.93	T13			$C_{24}H_{22}N_4O_8$	$[M+H]^-$	493.13649	493.13715	0.66	$[M+H]^+$	495.15104	495.15013	-0.91
8.16	T14			$C_{24}H_{22}N_4O_8$	$[M+H]^-$	493.13649	493.13712	0.63	$[M+H]^+$	495.15104	495.14999	-1.05
8.48	T15			$C_{24}H_{22}N_4O_8$	$[M+H]^-$	493.13649	493.13730	0.81	$[M+H]^+$	495.15104	495.15073	-0.31
9.14	T16			$C_{24}H_{22}N_4O_8$	$[M+H]^-$	493.13649	493.13716	0.67	$[M+H]^+$	495.15104	495.14991	-1.13

Table S3. List of the 2-acetamido-phenol trimers and their nitrosylated and nitrated derivatives detected in the bacterial exudate extracts *F49* of *A. aminovorans*. The corresponding chromatogram is shown on figure 5.

*t*R	Metabolite	Nr.	Neutral formula	ID level	Adduct ion	*m/z* det.	*m/z* calcd.	Error, mDa	ms2 HCD 30 eV
Identification of major consituents of the exudate extracts F49 and F51 (Method 1)									
3.11	2-acetamido-phenol	**2**	C8H9NO2	1	[M-H]-	150.05579	150.0555	0.29	150.0 (P, < 1), 109 (8), 108 (100), 107 (52), 91 (3), 81 (3)
3.57	*N*-(2-hydroxy-5-nitrosophenyl)acetamide	**6**	C8H8N2O3	2	[M-H]-	179.04587	179.04567	0.20	179.046 (P, < 1), 137 (46), 108 (6), 107 (100)
4.78	*N*-(2-hydroxy-5-nitrophenyl)acetamide	**5**	C8H8N2O4	1	[M-H]-	195.04079	195.04113	-0.34	195.041 (P, < 1), 153 (100), 124 (5), 123 (65), 108 (4)
4.96	*N*-(2-hydroxy-3-nitrophenyl)acetamide	**4**	C8H8N2O4	1	[M-H]-	195.04084	195.04113	-0.29	195.041 (P, < 1), 153 (44), 137 (5), 136 (100), 134 (6), 124 (7), 123 (100), 91 (59)
3.11	2-acetamido-phenol	**2**	C8H9NO2	1	[M+H]+	152.07042	152.07061	-0.19	152.1 (P, < 1), 111 (17), 110 (59), 93 (24), 92 (81), 65 (100)
3.57	*N*-(2-hydroxy-5-nitrosophenyl)acetamide	**6**	C8H8N2O3	2	[M+H]+	181.06085	181.06077	0.08	181.1 (P, < 1), 139 (6), 122 (3), 109 (100), 95 (5), 80 (10)
4.78	*N*-(2-hydroxy-5-nitrophenyl)acetamide	**5**	C8H8N2O4	1	[M+H]+	197.0553	197.05568	-0.38	197.1 (P, < 1), 155 (2), 138 (5), 126 (8), 110 (7), 109 (100), 80 (17)
4.96	*N*-(2-hydroxy-3-nitrophenyl)acetamide	**4**	C8H8N2O4	1	[M+H]+	197.05533	197.05568	-0.35	197.1 (P, < 1), 162 (2), 137 (12), 132 (5), 109 (15), 91 (10), 82 (100), 79 (5), 64 (10)
Incubation of bacteria, fungi & consortiums with 2-acetamido-phenol (Method 2)									
6.47	*N*-(2-hydroxy-5-nitrophenyl)acetamide glucoside	**9**	C14H18O9N2	3	[M-H]-	357.09455	357.09395	0.60	357.1 (P, < 1), 195 (100), 153 (30), 123 (1)
7.95	*N*-(2-hydroxy-5-nitrophenyl)acetamide sulfate	**11**	C8H8O7N2S	1	[M-H]-	274.99834	274.99794	0.40	275.0 (P, 2), 195 (100), 153 (17), 96 (2)
8.55	*N*-(2-hydroxy-5-nitrophenyl)acetamide	**5**	C8H8N2O4	3	[M-H]-	195.04112	195.04113	-0.01	195.0 (P, 35), 153 (100), 123 (5), 96 (1)
9.89	*N*-(2-hydroxy-5-nitrophenyl)acetamide glucoside-succinate	**10**	C18H22N2O12	3	[M-H]-	457.11069	457.11000	0.69	457.1 (P, < 1), 195 (100), 153 (17); in-source fragment 357 (P-100.01642, C4H4O3, 3.7 ppm)
6.47	*N*-(2-hydroxy-5-nitrophenyl)acetamide glucoside	**9**	C14H18O9N2	3	[M+H]+	359.10748	359.10851	-1.03	359.1 (P, < 1), 197 (37), 155 (100), 109 (3), 97 (3), 85 (20)
7.95	*N*-(2-hydroxy-5-nitrophenyl)acetamide sulfate	**11**	C8H8O7N2S	1	[M+H]+	277.01152	277.01250	-0.98	277.0 (P, < 1), 235 (6), 197 (6), 155 (100), 109 (15)
8.55	*N*-(2-hydroxy-5-nitrophenyl)acetamide	**5**	C8H8N2O4	3	[M+H]+	197.05539	197.05568	-0.29	197.0 (P, 2), 179 (2), 155 (100), 138 (1), 109 (40)
9.89	*N*-(2-hydroxy-5-nitrophenyl)acetamide glucoside-succinate	**10**	C18H22N2O12	3	[M+H]+	459.12405	459.12455	-0.50	not acquired
Exposure of kohlrabi seedlings to* N*-(2-hydroxy-5-nitrophenyl)- &*N*-(2-hydroxy-6-nitrophenyl)acetamide (Method 1)									
1.51	Thiosugar (SciFinder, CAS 174222-66-3)	**13**	C8H16O7S2	4	[M-H]-	287.02685	287.02647	0.38	287.0 (P, < 1), 79 (50), 75 (100, C2H3SO), 64 (10)
2.24	Glucoerucin	**14**	C12H23O9NS3	3	[M-H]-	420.04691	420.04621	0.70	420.1 (P, 17), 275 (40), 259 (91), 227 (38), 195 (27), 178 (100), 163 (14), 139 (39), 119 (44)
2.70	N-(2-hydroxy-6-nitrophenyl)acetamide glucoside	**15**	C14H18O9N2	3	[M-H]-	357.09455	357.09395	0.60	357.1 (P, < 1), 195 (3), 153 (100), 123 (5)
3.34	*N*-(2-hydroxy-6-nitrophenyl)acetamide	**7**	C8H8N2O4	2	[M-H]-	195.04129	195.04113	0.16	195.1 (P, < 1), 153 (26), 136 (5), 123 (100), 119 (12), 106 (15), 78 (6)
3.55	*N*-(2-hydroxy-5-nitrophenyl)acetamide glucoside	**9**	C14H18O9N2	3	[M-H]-	357.09455	357.09395	0.60	357.1 (P, < 1), 195 (42), 153 (100)
3.86	*N*-(2-hydroxy-6-nitrophenyl)acetamide acetylglucoside	**16**	C16H20O10N2	3	[M-H]-	399.10490	399.10452	0.38	399.1 (P, < 1), 195 (10), 153 (100), 123 (5)
4.21	Neoglucobrassicin	**17**	C17H22O10N2S2	3	[M-H]-	477.06527	477.06431	0.96	477.1 (P, < 1), 97 (100, HSO4), in-source diagnostic fragment 446.04693 (P - *OCH3, 80)
4.77	*N*-(2-hydroxy-5-nitrophenyl)acetamide	**5**	C8H8N2O4	1	[M-H]-	195.04132	195.04113	0.19	195.0 (P, < 1), 153 (100), 124 (5), 123 (65), 108 (4)
5.62	2-amino-3-nitrophenol	**18**	C6H6N2O3	1	[M-H]-	153.03069	153.03057	0.12	153.0 (P, < 1), 122 (100), 119 (12), 106 (12), 95 (20), 92 (10), 78 (15)
1.51	Thiosugar (SciFinder, CAS 174222-66-3)	**13**	C8H16O7S2	4	n.d.				
2.24	Glucoerucin	**14**	C12H23O9NS3	3	n.d.				
2.70	*N*-(2-hydroxy-6-nitrophenyl)acetamide glucoside	**15**	C14H18O9N2	3	[M+H]+	359.10846	359.10851	-0.05	359.1 (P, < 1), 197 (3), 155 (100), 137 (2), 97 (3), 85 (5); isolated in-source fragment 197: 197 (P, < 1
3.34	*N*-(2-hydroxy-6-nitrophenyl)acetamide	**7**	C8H8N2O4	2	[M+H]+	197.05562	197.05568	-0.06	197.0 (P, < 1), 155 (37), 137 (41), 107 (100), 95 (2), 82 (3), 79 (2)
3.55	*N*-(2-hydroxy-5-nitrophenyl)acetamide glucoside	**9**	C14H18O9N2	3	[M+H]+	359.10766	359.10851	-0.85	359.1 (P, < 1), 197 (3), 155 (100), 127 (4), 109 (32), 97 (9), 85 (27), 69 (8)
3.86	*N*-(2-hydroxy-6-nitrophenyl)acetamide acetylglucoside	**16**	C16H20O10N2	3	[M+H]+	401.11816	401.11773	0.43	not acquired
4.21	Neoglucobrassicin	**17**	C17H22O10N2S2	3	[M+H]+	479.07904	479.07886	0.18	not acquired
4.77	*N*-(2-hydroxy-5-nitrophenyl)acetamide	**5**	C8H8N2O4	1	[M+H]+	197.05563	197.05568	-0.05	197.1 (P, < 1), 155(2), 138 (5), 126 (8), 109 (100), 80 (19)
5.62	2-amino-3-nitrophenol	**18**	C6H6N2O3	1	[M+H]+	155.04500	155.04511	-0.11	155.1 (P, < 1), 138 (2), 126 (3), 108 (18), 107 (50), 96 (3), 90 (33), 80 (79), 72 (100)

Table S4. Overview of the LC-HR-MS and MSMS data of the metabolites detected and annotated in the bacterial extract of *A. aminovorans F49*, in bacterial and fungal extracts after incubation with **5**, and in kohlrabi seedlings exposed to **5** and **7**.

Table S5. Primers used for qPCR experiments

Gene	Locus identifier	Forward primer	Reverse primer
VTE1	At4g32770	ATTTGCGATGATGGCCGTAC	AACTCAAACCTTTCACCGCC
TPS 04	At1g61120	ATGGGAAGGAGAAGAGCTTAA	TTAGTAGAAGCATGGTGCGAAT
TPS 02	At4g16730	TAAAGAAGAGGTGAGGAAGAC	CTAGAAATAAGTTTAAGTTCT
TRYPS02	At5g54810	GTATCCCAATTCCCAACTTGTGT	AGCAGACACATGTAAGCAGACC
GLN1.1	At5g37600	CAATGAGGGAAGAAGGCGGT	CGCAACACCCCAAAGGAAAG
GLN1.2	At1g66200	CTTTCCTTTGGGGTGTTGCG	AGCTGGCCTCCTATCCTCAA
NIA1(NR1)	At1g77760	ACAAAGGCAAAGGCAACTTC	CCACATACATCTCGGTTTCGT
NIA2 NR2)	At1g37130	GCGTGGTGTCCCTCTCTG	TGATGCTCGTTCCGTATTTG
ACT2	At3g18780	CTTGCACCAAGCAGCATGAA	CCGATCCAGACACTGTACTTCCTT

Method 1: analysis of the exudate extracts, obtained from *A. aminovorans* (*F49*) / *P. polymixa* (*F51*) & incubation of kohlrabi seedlings with 5 and 7

Column: Acquity BEH C18 2.1x100mm (1.7 um, 130A)
Acquity HSS T3 2.1x100mm (1.7 um, 130A) was employed for confirmation of the identity of 3N-AAP *with* co-analysis of synthetic reference

Solvents: (A) H_2O + 0.1% HCOOH (*v/v*) Temp., °C: 30
(B) ACN + 0.1% HCOOH (*v/v*) Flow, uL/min: 450

Time, min	%A	%B	Curve
0	5	95	5
8	30	70	5
9	99	1	5
11	99	1	5
12	5	95	5
15	5	95	5

MS settings:

Resolution	70000
IT, ms	100
Microscans	1
AGC, charges	3e6
Polarity	+/-
Mass range	100 to 1200

MS2: data were collected continually for the masses of interest using inclusion list
default settings: CE=30 eV, R=17500, isolation width = 3 Da

LC-isCID/MS2: mass range *m/z* 50-300 at R=35000
in-source CID and MS2 were enabled at 30 eV with default settings
precursor ions of interest were continually isolated according to inclusion list

Method 2: analysis of the exudate extracts, obtained from incubation of different bacterial species with 5

Column: Kinetex C18 2.1x50mm (1.3um, 100A)

Solvents: (A) H_2O + 0.1% HCOOH (*v/v*) Temp., °C: 40
(B) ACN + 0.1% HCOOH (*v/v*) Flow, uL/min: 350

Time, min	%A	%B	Curve
0	5	95	5
10	26	74	5
13.5	95	5	5
16.5	95	5	5
17	5	95	5
20	5	95	5

MS settings: broadband LC-isCID/MS2 method, consisting of 4 measurement traces for (+)-ESI and (–)-ESI modes separately
R = 17500, AGC = 1e6 charges, IT=64 ms, 1 microscan and automatic precursor exclusion

	Mode	Range, *m/z*	CE, eV	Isolation, Da	
Trace 1	MS	70-750	-	full range	
Trace 2	MS2	70-750	30	4	top5 DDA
Trace 3	isCID	50-750	30	full range	
Trace 4	isCID/MS2	50-750	30	4	top5 DDA

Cycle time: 900 ms mass ranges for FS/MS2 and isCID/MS2 should be slightly different,
as the same mass range setting resulted in problems with data recording

Table S6. LC(MS) methods used for the analytical separation and purification of the bacterial extracts
